# Supplementary material for: Examining and Fine-tuning the Selection of Glycan Compositions with GlyConnect Compozitor
Source: Mol Cell Proteomics. 2020 Nov 25;19(10):1602–18. doi: 10.1074/mcp.RA120.002041 (PMC8014996; doi:10.1074/mcp.RA120.002041)

Supplementary Material

Category	Reference	URL
**Protein**	UniProtKB/Swiss-Prot DB	uniprot.org
**Tissue**	Uberon ontology	uberon.org
**Plant Tissue**	BRENDA ontology	brenda-enzymes.org/ontology.php?ontology_id=3
**Cell Type**	Cell ontology	cellontology.org
**Cell Component**	Gene ontology	geneontology.org
**Cell line**	Cellosaurus DB	web.expasy.org/cellosaurus/
**Disease**	Disease ontology	disease-ontology.org

Supp Table 1: Controlled vocabularies and ontologies used in the GlyConnect database

List of 82 IgG potential compositions:

Hex:3 HexNAc:2, Hex:3 HexNAc:2 dHex:1, Hex:3 HexNAc:3, Hex:3 HexNAc:3 dHex:1, Hex:3 HexNAc:4, Hex:3 HexNAc:4 dHex:1, Hex:3 HexNAc:5, Hex:3 HexNAc:5 dHex:1, Hex:3 HexNAc:6, Hex:4 HexNAc:2, Hex:4 HexNAc:2 dHex:1, Hex:4 HexNAc:3, Hex:4 HexNAc:3 dHex:1, Hex:4 HexNAc:3 dHex:1 NeuAc:1, Hex:4 HexNAc:3 dHex:2, Hex:4 HexNAc:3 NeuAc:1, Hex:4 HexNAc:4, Hex:4 HexNAc:4 dHex:1, Hex:4 HexNAc:4 dHex:2, Hex:4 HexNAc:4 dHex:1 NeuAc:1, Hex:4 HexNAc:4 dHex:3, Hex:4 HexNAc:4 NeuAc:1, Hex:4 HexNAc:5, Hex:4 HexNAc:5 dHex:1, Hex:4 HexNAc:5 dHex:1 NeuAc:1, Hex:4 HexNAc:5 dHex:2, Hex:4 HexNAc:5 NeuAc:1, Hex:5 HexNAc:2, Hex:5 HexNAc:3, Hex:5 HexNAc:3 dHex:1, Hex:5 HexNAc:3 dHex:1 NeuAc:1, Hex:5 HexNAc:3 NeuAc:1, Hex:5 HexNAc:4, Hex:5 HexNAc:4 dHex:1, Hex:5 HexNAc:4 dHex:1 NeuAc:1, Hex:5 HexNAc:4 dHex:1 NeuAc:2, Hex:5 HexNAc:4 dHex:2, Hex:5 HexNAc:4 dHex:2 NeuAc:1, Hex:5 HexNAc:4 dHex:3, Hex:5 HexNAc:4 NeuAc:1, Hex:5 HexNAc:4 NeuAc:2, Hex:5 HexNAc:5, Hex:5 HexNAc:5 dHex:1, Hex:5 HexNAc:5 dHex:1 NeuAc:1, Hex:5 HexNAc:5 dHex:1 NeuAc:2, Hex:5 HexNAc:5 dHex:2, Hex:5 HexNAc:5 dHex:3, Hex:5 HexNAc:5 NeuAc:1, Hex:5 HexNAc:5 NeuAc:2, Hex:6 HexNAc:2, Hex:6 HexNAc:3, Hex:6 HexNAc:3 dHex:1, Hex:6 HexNAc:3 dHex:1 NeuAc:1, Hex:6 HexNAc:3 NeuAc:1, Hex:6 HexNAc:4, Hex:6 HexNAc:4 dHex:1, Hex:6 HexNAc:4 dHex:1 NeuAc:1, Hex:6 HexNAc:4 NeuAc:1, Hex:6 HexNAc:4 NeuAc:2, Hex:6 HexNAc:5, Hex:6 HexNAc:5 dHex:1, Hex:6 HexNAc:5 dHex:1 NeuAc:1, Hex:6 HexNAc:5 dHex:1 NeuAc:2, Hex:6 HexNAc:5 dHex:2, Hex:6 HexNAc:5 NeuAc:1, Hex:6 HexNAc:5 NeuAc:2, Hex:6 HexNAc:5 NeuAc:3, Hex:6 HexNAc:6, Hex:6 HexNAc:6 dHex:1, Hex:6 HexNAc:6 dHex:1 NeuAc:2, Hex:7 HexNAc:2, Hex:7 HexNAc:3, Hex:7 HexNAc:3 dHex:1, Hex:7 HexNAc:4, Hex:7 HexNAc:4 dHex:1, Hex:7 HexNAc:5 dHex:1, Hex:7 HexNAc:6, Hex:7 HexNAc:6 NeuAc:1, Hex:7 HexNAc:7, Hex:8 HexNAc:2, Hex:9 HexNAc:2, Hex:10 HexNAc:2.

Supplemental figure legends

Supp Figure 1: Examples of protein large N-glycomes and connecting role of virtual nodes

The glycome size does not correlate with an increase in virtual nodes. (A) The N-glycome of human thrombospondin-1 is composed of 83 compositions but only two virtual nodes are needed to fully connect corresponding compositions (P07996). (B) In contrast, the N-glycome of non-recombinant erythropoietin (P01588) is composed of 58 compositions but sixteen virtual nodes are needed to fully connect corresponding compositions.

Supp Figure 2: Differential connectivity of the same node in two N-glycomes

A composition node can be central in one graph and a terminal leaf in another. Its structuring role in the graph is then different. (A) In the N-glycome graph of human erythropoietin (P01588) H6N5F1S2 connects two regions of the graph. Its removal would cause the collapse of parts and create two separate clusters. (B) In the N-glycome graph of human decorin (P07585) H6N5F1S2 is pre-terminal. Its removal would only result in isolating the leaf node H6N5F1S3.

Supp Figure 3: Differential roles of same node in two extracellular matrix (ECM) proteins

A composition node can be virtual in one graph and real in another. (A) H4N4F2 is a virtual node (9 cyan links and 11 orange links) in the N-glycome graph of human decorin (P07585) (B) H4N4F2 is a regular node (10 cyan links and 12 orange links) in the N-glycome graph of human thrombospondin-1 (P07996) (C) when virtual nodes are omitted in the N-glycome graph of human decorin (P07585) H4N4F1 and H4N4F3 are remote (D) when virtual nodes are included in the N-glycome graph of human decorin (P07585) then H4N4F2 occurs and it connects logically H4N4F1 and H4N4F3.

Supp Figure 4: Connectivity in the graph of human decorin N-glycome

Path highlighting reveals the contribution of a node to a graph as seen in the N-glycome of human decorin (P07585). (A)The H5N4F1 node is a connector between two areas of the graph (17 cyan links and 12 orange links) (B) The H5N4F2S1 node is terminal (22 cyan links)

Supp Figure 5: Comparison of conserved glycosylated asparagine in two highly similar human proteins

Asn-72 is a conserved glycosite in 89.5% similar alpha-1 acid glycoprotein 1 (P02763) and alpha-1 acid glycoprotein 2 (P19652). However, the glycome comparison of the two respective glycosites shows the total inclusion of the latter in the former (no red nodes). The corresponding bar plots show differences mainly in proportions of neutral and sialylated compositions.

Supp Figure 6: CHO cell N-glycome with and without virtual nodes

The N-glycome of the generic CHO cell line (CVCL_0213) includes 79 compositions. They are mapped in Compozitor with 16 virtual nodes in a single graph that only leaves N1F1 isolated since this composition is particularly small, as shown in (A) and without virtual nodes creating three clusters and leaving three nodes isolated, as shown in (B).

Supp Figure 7: Mascot composition file with and without virtual nodes as submitted in Ref.3

A set of 205 potential compositions was estimated from details of (3) where Mascot was used for intact glycopeptide identification. The mesh-like regular structure of the graph reflects the systematic approach for generating compositions (A). This regularity is confirmed by the very low number of virtual nodes needed to close the graph (B).

Supp Figure 8: GPQuest composition file with and without virtual nodes as submitted in Ref 26 and 27

A file of 181 compositions was communicated by the authors of (26)(27) and input in Compozitor. The outline of the graph almost closed with 27 virtual nodes (A) is similar to many biological networks where several extensions stem from central nodes. A few clusters of large compositions (#H >10) do not fit in. Node scattering is greater when virtual nodes are not included (B).

Supp Figure 9: Byonic composition file with and without virtual nodes as submitted in Ref 4 and 28

305 of default 309 compositions provided with the Byonic search engine were input in Compozitor. The outline of the graph almost closed with 35 virtual nodes (A) is similar to many biological networks where several extensions stem from central nodes. A few clusters of large compositions (#H >10) do not fit in. Node scattering is greater when virtual nodes are not included (B).

Supp Figure 10: Comparison of node connectivity in Figure 6B graph with and without virtual nodes

Neighbourhood of H6N7 in (A) an excerpt of the graph shown in Figure 6B and (B) its counterpart with no virtual nodes. Each node is labelled with the number of outgoing (cyan) and incoming (orange) paths connecting it to other nodes in the graph. Yellow nodes corresponding to identified compositions in the secretome of endothelial cells are particularly impacted by the introduction of virtual node H5N7.

GlyConnect Compozitor

Supp Figure S1

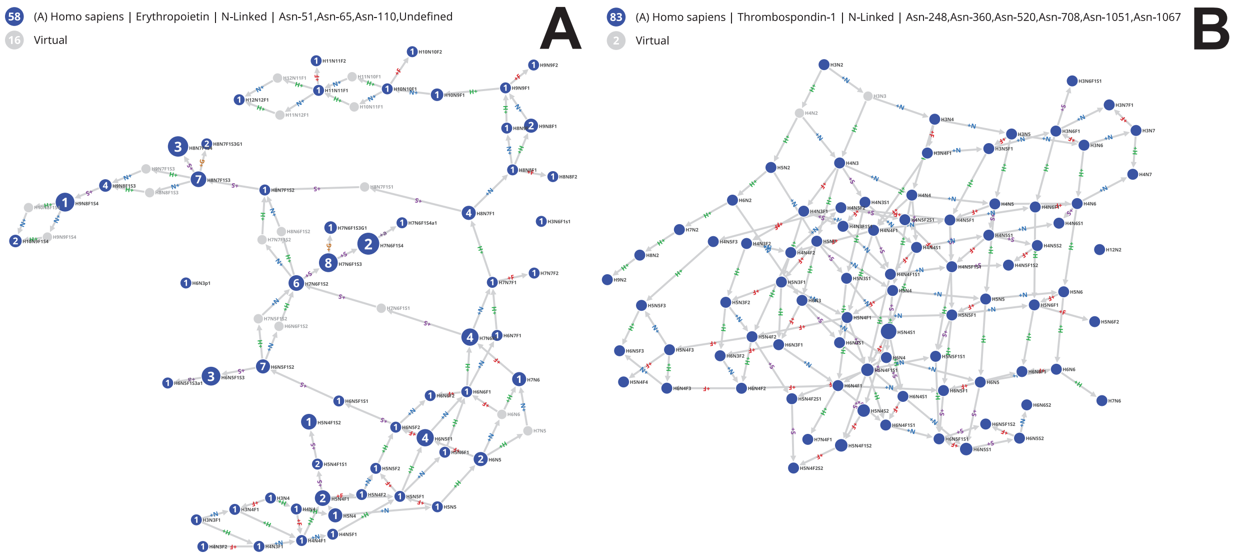

Supp Figure S2

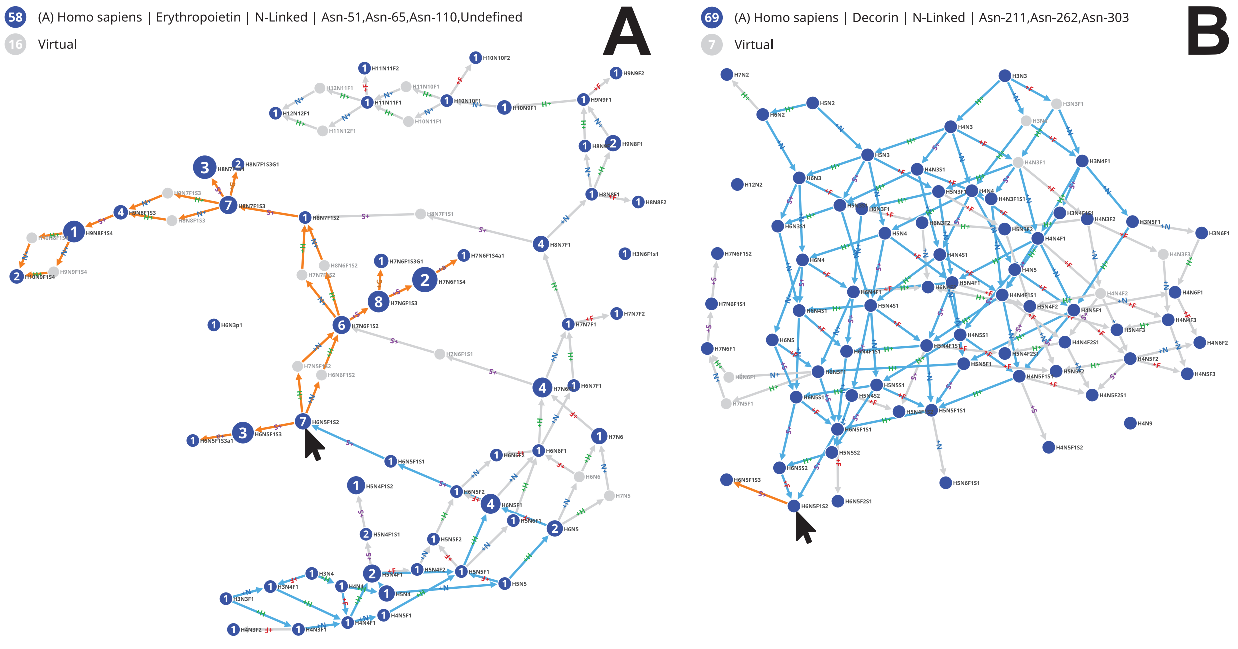

GlyConnect Compozitor

Supp Figure S3

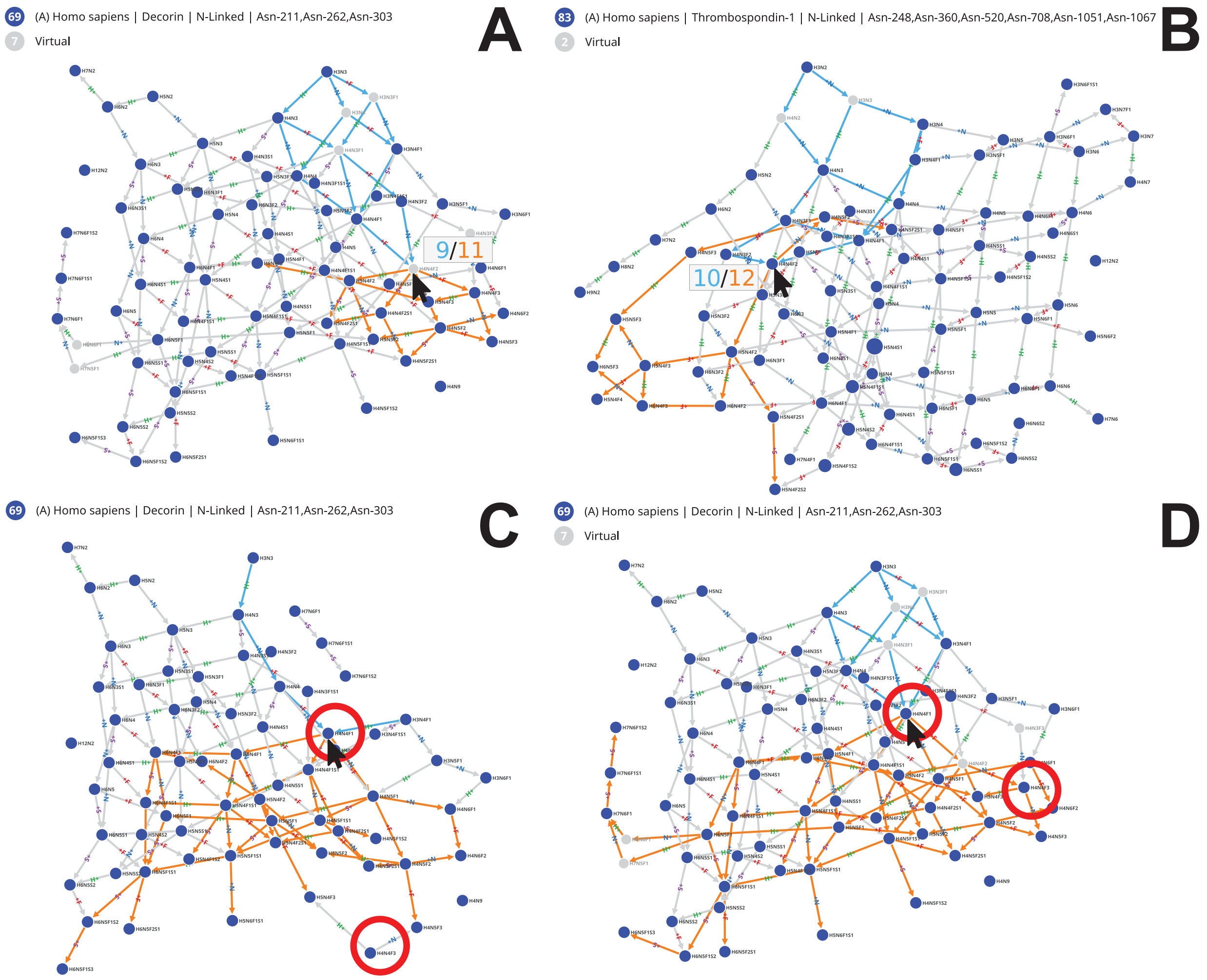

Supp Figure S4

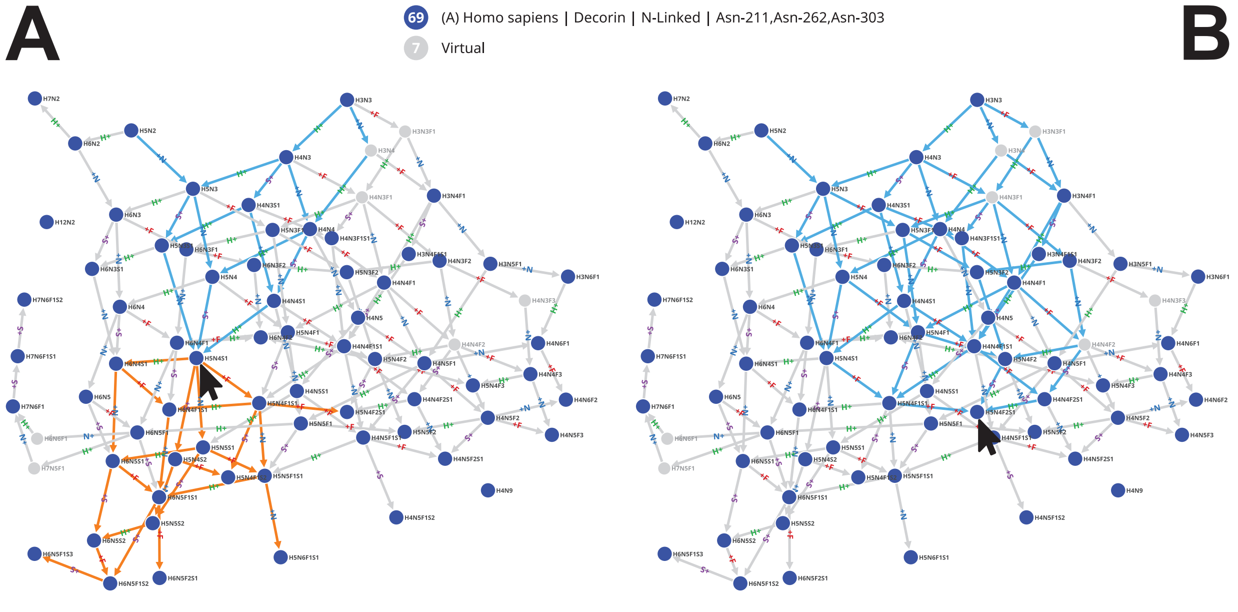

Supp Figure S5

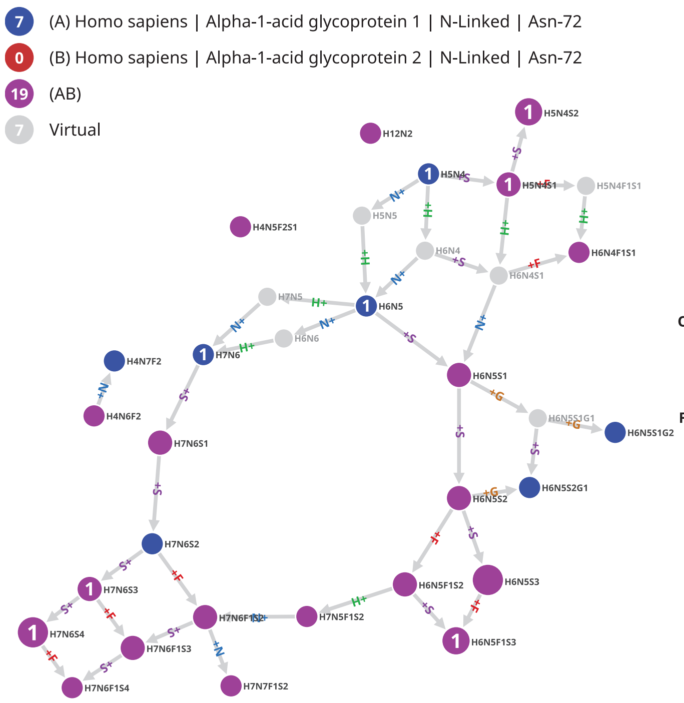

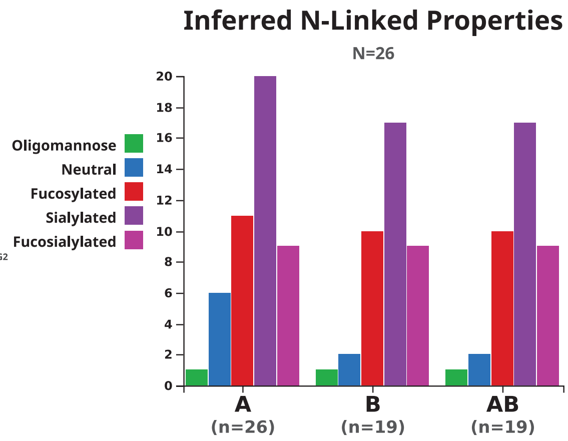

Supp Figure S6

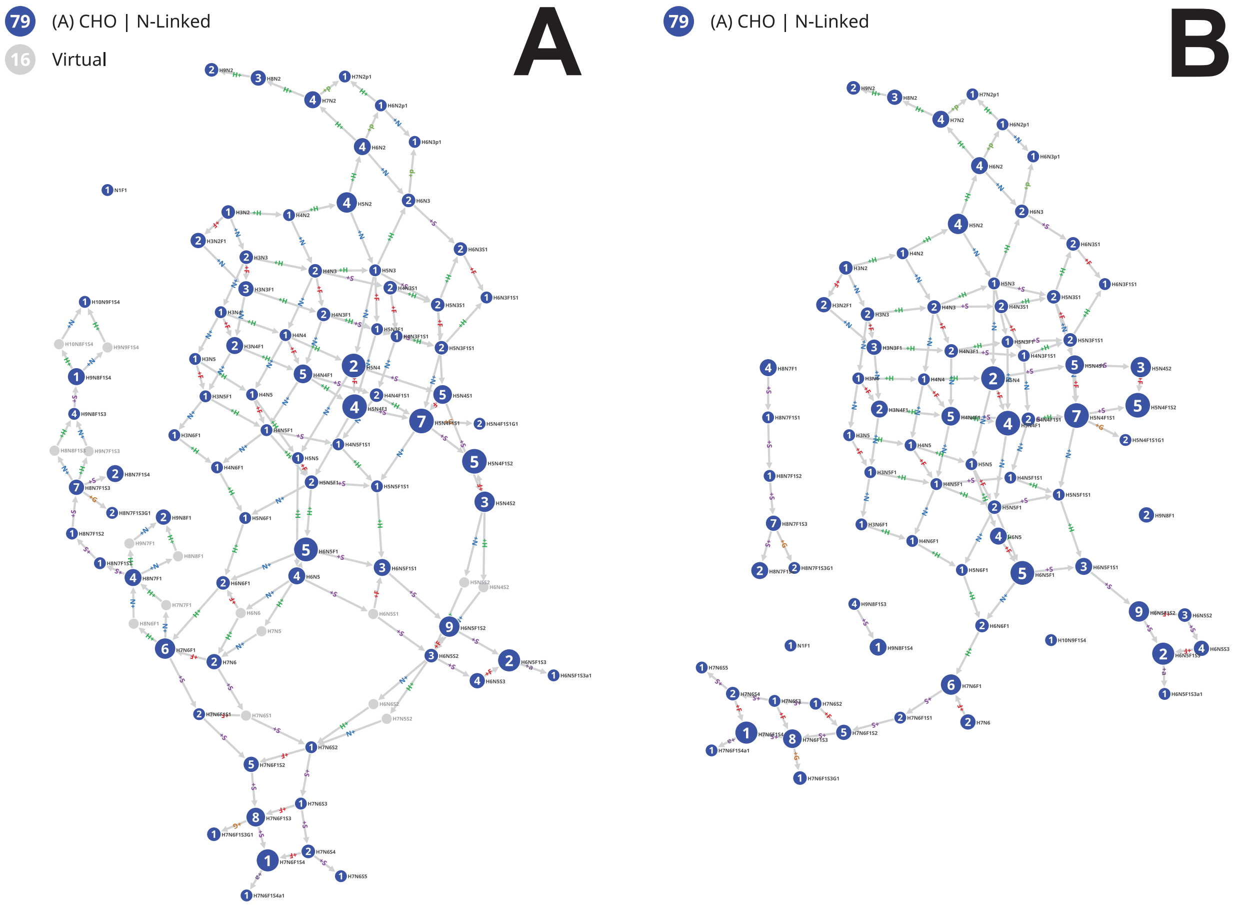

Supp Figure S7

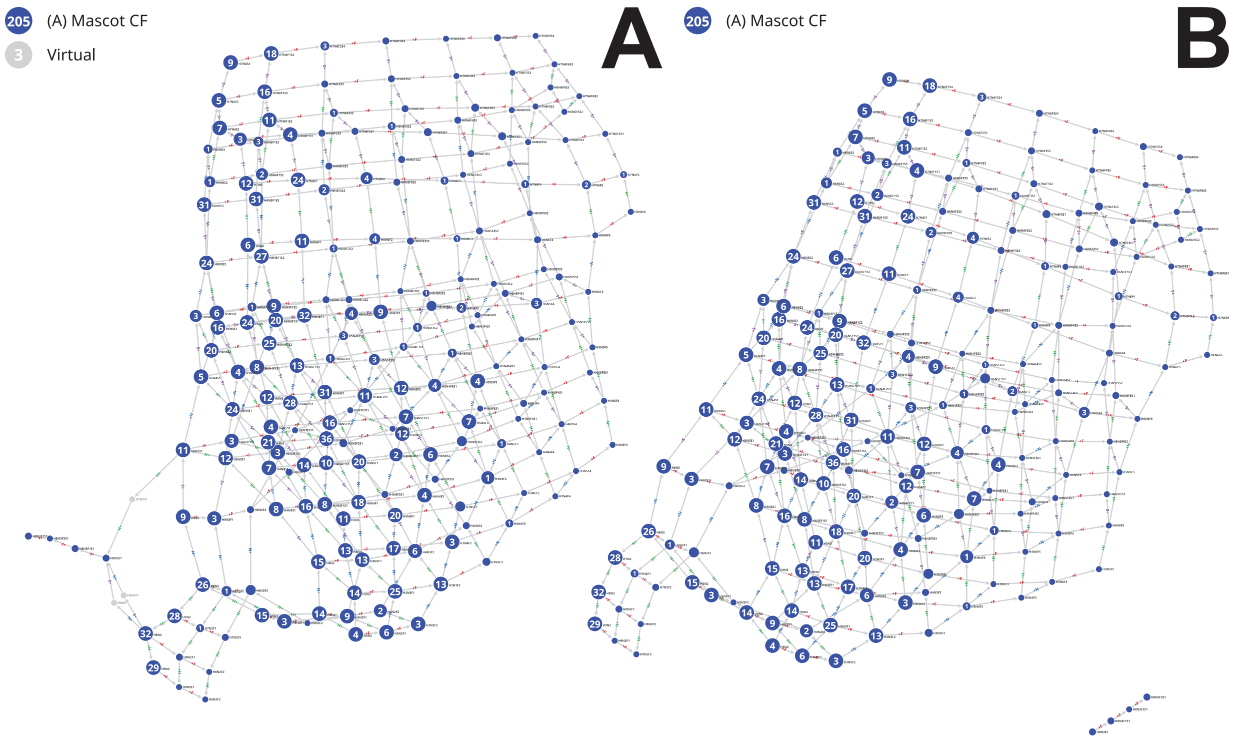

Supp Figure S8

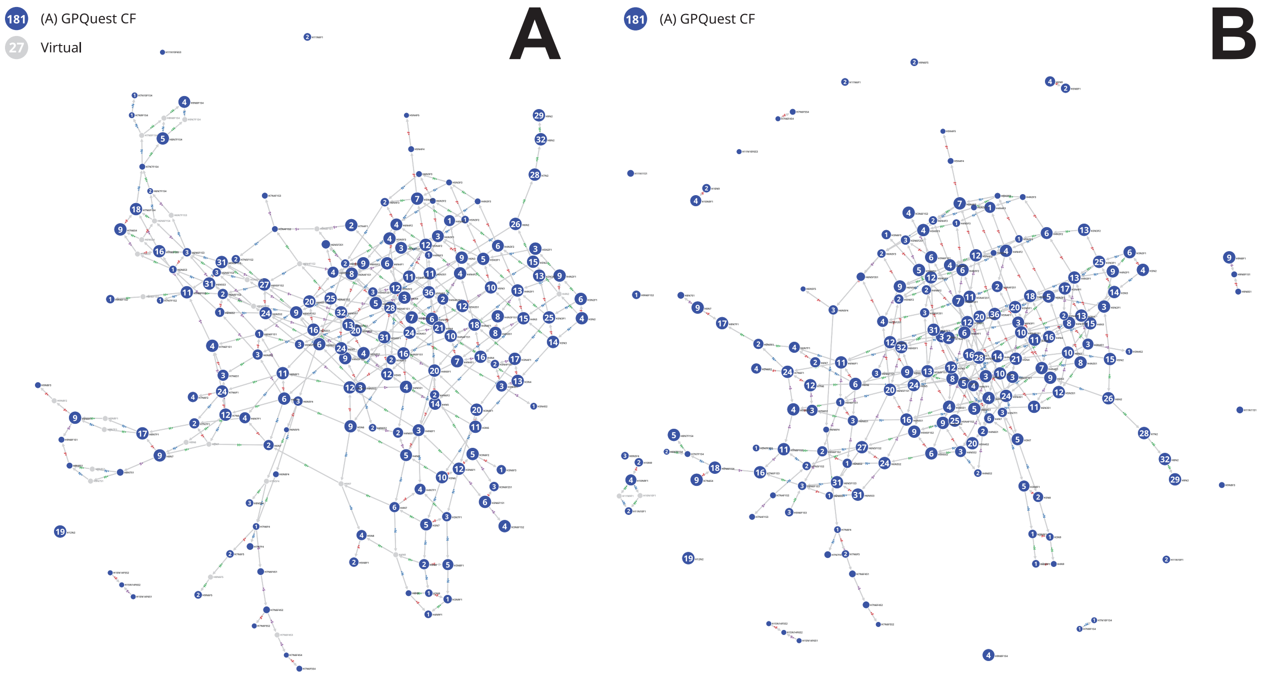

Supp Figure S9

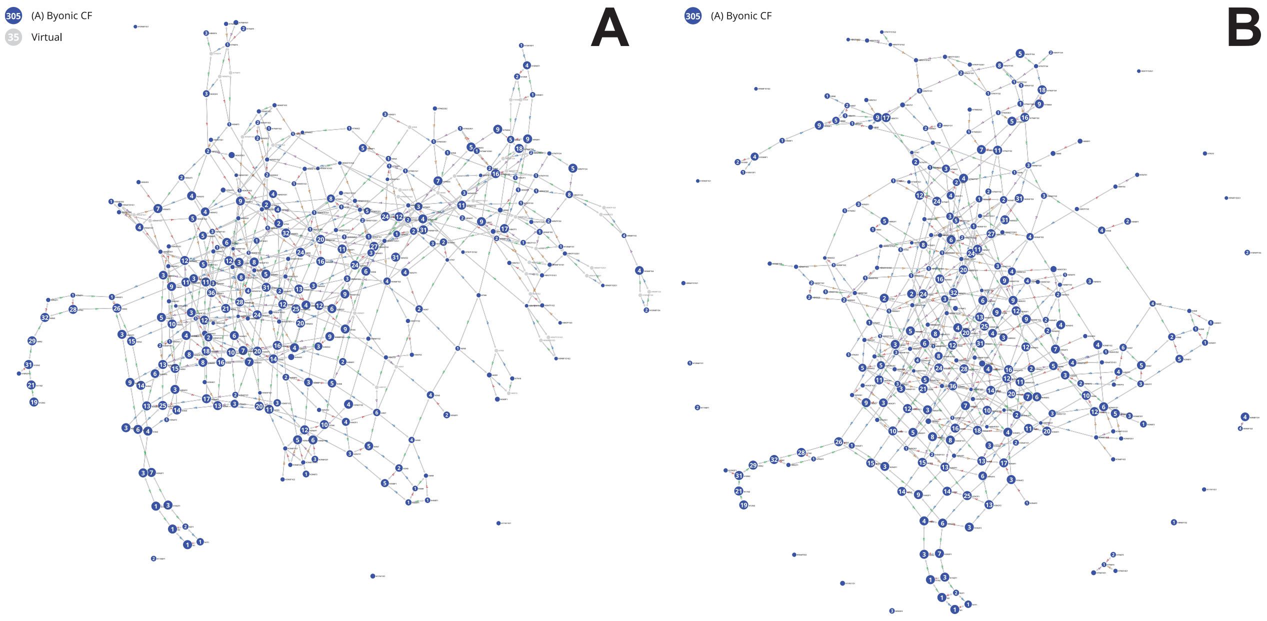

Supp Figure S10

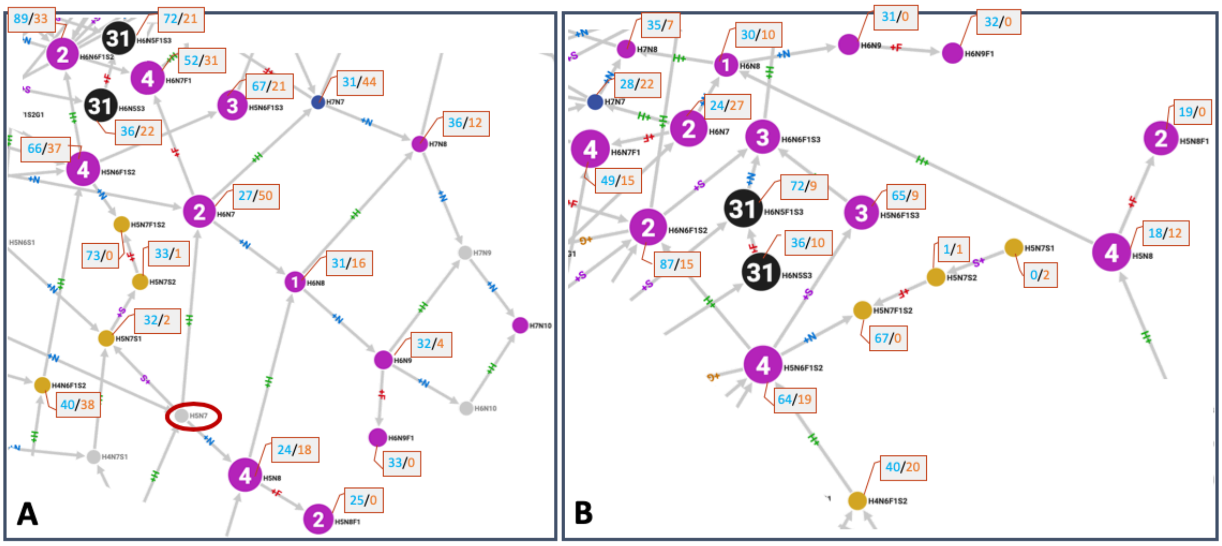

Supplement: Supplementary file 1 [file mmc1.pdf]
